# Regional variation in coronary angiography rates: the association with supply factors and the role of indication: a spatial analysis

**DOI:** 10.1186/s12872-022-02513-z

**Published:** 2022-02-26

**Authors:** Julia Frank-Tewaag, Julian Bleek, Christian Günster, Udo Schneider, Dirk Horenkamp-Sonntag, Ursula Marschall, Sebastian Franke, Kathrin Schlößler, Norbert Donner-Banzhoff, Leonie Sundmacher

**Affiliations:** 1 Technische Universität München, Uptown München Campus D, Georg-Brauchle-Ring 60/62, 80992 München, Germany; 2 Institute for Medical Information Processing, Biometry, and Epidemiology (IBE), Ludwig-Maximilians-University Munich, Munich, Germany; 3 Pettenkofer School of Public Health, Munich, Germany; 4 AOK Bundesverband, Berlin, Germany; 5 Wissenschaftliches Institut der AOK (WIdO), Berlin, Germany; 6 Techniker Krankenkasse (TK), Hamburg, Germany; 7 BARMER, Wuppertal, Germany; 8 Department of Family Medicine / General Practice, Ruhr-University Bochum, Bochum, Germany; 9 Department of Family Medicine / General Practice, Philipps-University of Marburg, Marburg, Germany

**Keywords:** Coronary heart disease, Coronary angiography, Clinical practice variation, Indication, Regional analysis

## Abstract

**Background:**

Coronary angiographies (CAs) are among the most common diagnostic procedures carried out in German hospitals, and substantial regional differences in their frequency of use have been documented. Given the heterogeneity with regard to the expected benefits and the varying scope for discretion depending on the indication for the procedure, we hypothesized that the observed variation and the association of need and supply factors differs by indication for CA.

**Methods:**

We investigated the correlation between supply factors and the regional rates of CAs in Germany while controlling for need using spatial‐autoregressive error models (SARE) and spatial cross-regressive models with autoregressive errors (SCRARE). The overall rates of CAs and the rates in specific patient subgroups, namely, patients with and without myocardial infarction (MI), were calculated based on a comprehensive set of nationwide routine data from three statutory health insurances at the district level.

**Results:**

Although little variation was found in cases with MI, considerable variation was seen in the overall cases and cases without MI. The SARE models revealed a positive association between the number of hospitals with a cardiac catheterization laboratory per 10,000 population and the rates of overall cases and cases without MI, whereas no such relationship existed in cases with MI. Additionally, an association between regional deprivation and the rates of CAs was found in cases with MI, but no such association was seen in cases without MI.

**Conclusions:**

The results supported the hypothesis that the relative association of need and supply factors differed by the indication for CA. Although the regional differences in the frequency of use of CAs can only be explained in part by the factors examined in our study, it offers insight into patient access to and the provision of CA services and can provide a platform for further local research.

**Supplementary Information:**

The online version contains supplementary material available at 10.1186/s12872-022-02513-z.

## Introduction

Coronary angiography (CA) is an invasive diagnostic procedure that aids in making the decision between conservative treatment only or a revascularization procedure, namely, percutaneous coronary intervention (PCI) or bypass surgery (CABG). Current guidelines emphasize the role of CA in preparing for treatment while limiting purely diagnostic indications [1–4]. Regional variation in the utilization of CA and PCI has been documented across different countries and health systems [5–14]. CAs and PCIs are among the most common diagnostic procedures carried out in German hospitals [15], and the rates of these procedures in Germany have been deemed to be rather high in comparison with those documented in other European health systems [16]. In addition, substantial regional differences in the frequency of use of CAs and PCIs have been documented in Germany [17, 18]. It has therefore been discussed repeatedly whether these findings truly reflect differences in medical need [16–18].

Some regions, for example, may exhibit higher rates of CAs because of greater demand in these localities. Demand, in the broadest sense, reflects the patient-related factors that influence the actual or perceived need for a procedure. These factors include the incidence of a treatable disease, the rate at which the disease is detected prior to the procedure and the willingness of patients to undergo the procedure [19]. However, it may be the case that factors other than patients’ needs or preferences are driving treatment decisions, particularly when the procedure in question leaves room for discretion. Piedmont et al. [18] found an almost linear association between the regional rate of CAs in the districts of the German federal state of Saxony-Anhalt and the number of cases without therapeutic consequences. The authors noted that they were unable to satisfactorily assess the influence of regional supply structures on the frequency of use, but they pointed out that the wide range in the proportion of procedures without therapeutic consequences indicates an influence of supply-related factors. The number of catheterization facilities in Germany has increased in recent years, and needs-based large equipment planning in hospitals (§ 122 SGB V) was once envisaged but was cancelled without replacement in 1997 [20].

Wennberg et al. [21] suggested a relationship between the availability and quality of evidence to support a particular intervention and the variation in its use. When there is greater uncertainty about the evidence base for a procedure, there is more likely to be variation. The authors distinguished among three categories of care. Effective care comprises services that are supported by a well-established evidence base for their efficacy. Preference-sensitive care includes services where at least two valid alternative strategies for action are available, and the decision involves a trade-off because the risks and benefits of the options differ. Supply-sensitive care occurs primarily when there is a broad scope for decision-making due to a lack of evidence and guidelines to inform best practice or greater medical uncertainty [21, 22]. CAs and PCIs are ideal for the study of medical practice variations because they exhibit considerable heterogeneity with regard to their benefits across different patient groups, and the evidence and appropriateness criteria provide considerable opportunity for discretionary judgment depending on the indication for the procedure. For patients presenting with symptoms of acute coronary syndrome (ACS) or myocardial infarction (MI), the benefits of PCI are well established [23, 24]. In patients with chronically stable coronary heart disease (CHD), the decision is much less clear-cut [25, 26]. In these non-acute situations, the use of elective CA and PCI is preference sensitive because alternative treatment strategies exist, and the decision to undergo the procedure involves a trade-off between the risks and benefits. Owing to the broader scope for decision-making and the accompanying uncertainty in these circumstances, the available supply capacity may exert an influence on utilization.

Given the differences, we hypothesized that the observed variation and the possible association of need and supply factors differ by indication for CA. We therefore aimed to investigate regional variation in the rates of CAs and the correlation between supply factors and the regional rates of the procedure in patients with and without MI in Germany while controlling for the actual need. To this end, the overall rates of CAs and the rates in specific patient subgroups were calculated based on a unique, comprehensive set of nationwide regional routine data from statutory health insurances. Regional differences in the observed rates were scrutinized in small-area analyses at the district level with empirical Bayes (EB) smoothing to account for variance instability resulting from the differing sample sizes.

## Methods

### Data and study population

The analysis was based on a comprehensive set of linked nationwide billing data from three statutory health insurances for the years 2014 to 2016, which equated to a total insured population of almost 42 million. The sample cohort included patients who were aged 18 years or older and who underwent CA in 2016 in the hospital on an inpatient or outpatient basis (OPS 1–275.0 up to 1–275.5). In addition, because not only hospitals play a role in the provision of the procedure in Germany, patients who underwent CA in an office-based practice (EBM 34291) were also included in the sample. Furthermore, to be considered, patients needed to be insured for at least 360 days. Patients with a shorter insurance period were only considered for the analysis if the reduction in the duration of insurance occurred because the patients died at the time of the CA procedure or after. Insured individuals who switched their insurance between 2014 and 2016 and patients without details of their place of residence or those with implausible information were excluded from the analysis. We carried out the analysis at the billing case level. If a patient had multiple recorded procedures within a billing case, they were only counted once. Several treatment cases per patient on the same day of treatment were also considered as one case.

### Patient subgroups by indication

The patient subgroups by indication for CA were determined by means of the main inpatient diagnosis, an outpatient hospital diagnosis or a secured office-based diagnosis in the treatment case of the CA. In the main analysis, we present the overall rates of CAs, the rates of cases with MI (ICD-10: I21.* or I22.*) and the rates of cases without MI. This allowed for comparison of the rates in a situation of effective care (MI) in which a strong evidence base for efficacy guides the treatment decisions, as opposed to situations where greater uncertainty is present (cases without MI). To assess the plausibility of the results, in a sensitivity analysis, we extended the definition of an acute indication to ACS (ICD-10: I21.*, I22.*, I20.0 or I20.1). Again, the rates of cases without ACS and the rates of cases with ACS were examined. Last, cases who were treated for stable CHD or chest pain (CP) (ICD-10: I20.8, I20.9, I25.0, I25.1, I25.5, I25.8, I25.9 or R07.*) were included in the sensitivity analysis.

### Geographical assignment

The insured patients in the study sample were assigned to a geographical location based on their district of residence, not the treatment locations. In Germany, a district is an administrative unit between federal states and the local municipal levels. The advantage of this allocation was that a distortion of the results to the detriment of the independent cities was prevented, and the factors considered in our analysis were measured at the place of residence. The spatial allocation was based on the official district key (area status on 31st of December 2016), taking into account the territorial reform in Lower Saxony on 1st of November 2016. We used the information available at the time of the first procedure in 2016; if the information was missing, the last available information on the patient’s place of residence was considered.

### Rates, standardization and assessment of variation

The rates were calculated using the total insured populations of the three statutory health insurances as the denominator. To address the variance instability resulting from the differences in population size, we applied EB smoothing [27]. We calculated the rates per 10,000 population standardized for differences in age and sex by means of the direct and indirect method based on the reference population for Germany as of the reporting date 31.12.2015. We measured the relative degree of variation using the coefficient of variation (COV) and the systematic component of variation (SCV) [28]. In general, SCV values above 5 are indicative of high variation, whereas SCV values above 10 point toward very high variation [19].

### Empirical model

#### Dependent variable

We use the crude unadjusted CA rate as the dependent variable in the empirical model because the use of standardized rates as the outcome may lead to biased estimates when the relationship between the standardization parameters, in this case age and sex, and other explanatory variables is not taken into account [29, 30].

#### Independent variables

To incorporate the demographic risk structure of the regional population in each district, we included the proportion of residents in each age-sex group in our model. Because there were 12 age-sex groups, 11 independent variables were used in the regression model, and the age-sex group ‘females under the age of 40 years’ constituted the reference group. The actual occurrence of treatable disease and the rate at which it was detected was unknown, but it could be approximated in the routine data through the diagnosed prevalence of CHD in the districts. We estimated the diagnosed CHD prevalence in the routine data as the proportion of individuals in the total sample of insured with a confirmed diagnosis of CHD (ICD-10: I20–I25, an inpatient primary or a secondary diagnosis or a confirmed office-based diagnosis in at least two quarters) during 2016. In addition, diagnosed risk factors, namely, type II diabetes and hypertension, were considered as variables. However, the variables showed a high correlation with the estimated diagnosed CHD prevalence; they were not included in the model to avoid problems associated with multicollinearity. Other risk factors related to lifestyle, such as smoking, cannot be derived from routine data. However, it has been shown that these risk factors are highly correlated with deprivation at the district level. For this reason, the German Index of Social Deprivation (GISD) 2012, which has been validated in patient groups with CHD, was incorporated as an additional factor to mediate the residual morbidity risks that remained after accounting for the disease prevalence [31]. In addition, it must be noted that the perceived need and preference of the patients could not be estimated. However, these factors were modeled as unobserved heterogeneity. To assess the impact of regional health care supply, we included information on the availability of hospitals with cardiac catheterization laboratory facilities per 10,000 population in the model. This information was extracted from the hospitals’ quality reports [32]. The data did not contain information on office-based facilities apart from those listed in the reports as working in collaboration with a hospital. Additionally, the number of catheterization laboratories within a given facility was unknown.

### Spatial model

The estimation model was formulated as a generalized, spatial, two-stage, least square model with spatial‐autoregressive disturbances [33] based on the spatial models of Cliff and Ord (1973) [34]. We assumed that a spatial error model, as opposed to a spatial lag model, was suitable to describe the process underlying our data because we believe that it is unlikely that the error term is distributed independently across the districts. The spatial error model was consistent with the assumption that spatial correlation arises from the geographical concentration of unobservable factors, such as unmeasured health status, risk factors or patient preference, or in a situation where exogenous shocks in one district impinge on neighboring districts [35, 36]. We did not assume that the CA rate in one district was directly affected by the rate in another district and therefore discarded the spatial lag model. Regional spillover effects might be present if the use of health services was measured in the district of the service provision. However, because we measured the rates at the patients’ places of residence and not the providers’ locations, such spillover should not be of relevance. Our estimation Model A was therefore simplified to a spatial‐autoregressive error (SARE) model, in which only the error terms were spatially correlated [37]. The definition of the individual districts as the catchment area for the supply of medical services might be regarded as too small to accurately reflect the supply of CAs. Therefore, our estimation Model B was formulated as a spatial cross-regressive model with autoregressive errors (SCRARE) [38], which included a spatial lag of the independent variable measuring the availability of hospitals with cardiac catheterization laboratory facilities. This approach took into account that the supply in neighboring districts also affects the rates of CAs. To verify the theoretical consideration and presence of spatial autocorrelation and to select the appropriate model for estimation, we estimated ordinary least square (OLS) regression models and performed the Lagrange Multiplier test [39]. In both models, the weight matrix that reflected the relationship between spatial units was a queen contiguity matrix, which defined neighbors as sharing a common edge or a common vertex [27]. To allow for a straightforward interpretation of the model parameters, the spatial weighting matrix was normalized. Different normalization methods make different assumptions about the spatial interdependence of observations. A row-standardized spatial weights matrix implies that every region is subject to the same total amount of influence from all other regions [40]. Unless this implicit assumption is clearly suggested by economic theory, row normalization should not be applied [41, 42]. Felder and Tauchmann [43] that it is debatable whether the assumption that spatial interdependence is of equal relevance to all regions holds in the case of the German administrative districts because the districts, as spatial units, vary with respect to their interlinkage with the rest of the country and in terms of their remoteness. They proposed eigenvalue normalization [42], which allows for spatial dependence to be differently important across observations. In the present analysis, we applied both row- (*Wr*) and eigenvalue-normalization (*We*) and contrasted the results obtained under the diverging assumptions. The analyses were performed in Stata SE16 and GeoDa.

## Results

### Study sample

The dataset comprised nationwide linked billing data from the statutory health insurers AOK, BARMER and Techniker Krankenkasse from approximately 42.5 million individuals. In 2016, a total of 379,625 patients in the sample dataset underwent CA at least once. Of these patients, 269 (0.07%) were excluded because of incomplete or implausible information. For 584 patients (0.15%), information on the patients’ districts of residence was missing at the time of the first procedure in 2016; the last available information was considered for the geographic assignment.

### Frequency, characteristics and regional CA rates

In total, in 2016, 379,356 patients in our analysis sample had undergone CA at least once. Expressed as the number of cases (hospital or office-based practice visits with at least one CA procedure), this amounted to 425,163 procedures among 41,739,344 (1.02%) insured patients. Extrapolated to the total German population, this resulted in an absolute number of 753,135 patients who underwent CA at least once and 844,771 CA cases. Table 1 shows the frequencies of CA and revascularization procedures (PCI or CABG) by health care sector and the subgroups by treatment diagnoses in the main analysis. The majority of CAs were carried out in a hospital setting and in cases without MI. An intervention was performed in more than 40% of cases. Some 75,542 (17.77%) cases were treated for MI, of which over 99% were treated in an inpatient hospital setting (see Additional file 1: Table A2 for the sensitivity analysis).

**Table 1:** Frequency and characteristics of cardiac catheterization by treatment setting

		Hospital		Office-based practice
		Inpatient hospital	Outpatient hospital	
Cases	n (column %)	N (row %)		
CA	425,163	369,882 (87.00%)	19,317 (4.54%)	35,964 (8.46%)
PCI*	168,418 (39.61%)	163,503 (97.08%)	118 (0.07%)	4797 (2.85%)
CABG*	4642 (1.09%)	4642 (100.00%)	0 (0.00%)	0 (0.00%)
Treatment diagnosis**				
Cases with MI	75,542 (17.77%)	74,983 (99.26%)	62 (0.08%)	497 (0.66%)
Cases without MI	349,621 (82.23%)	294,899 (84.35%)	19,255 (5.51%)	35,467 (10.14%)

CA, coronary angiography; CABG, coronary artery bypass graft; MI, myocardial infarction; PCI, percutaneous coronary intervention

*In the same treatment case

**Inpatient main hospital diagnosis, outpatient hospital diagnosis, confirmed ambulatory diagnosis in treatment case

Table 2 summarizes the crude and adjusted CA rates and the measures of variation for the cases overall and the cases without MI and with MI. At the district level, the direct standardized rate (DSR) for the overall cases ranged from 46.79 to 229.19, with a mean of 102.69 cases per 10,000 population. The observed variation in the overall rate of CA cases showed a COV of over 25% and a SCV over 6, which suggests high variation. When looking at the subgroups by treatment diagnosis, the difference in the extent of variation at the level of the 401 districts became apparent. The rate of cases with MI varied 3.53-fold, and the rate of those without MI varied 7.78-fold. The variation measured in the rate of CA cases with MI was relatively low (COV = 19.5 and SCV = 3.37) in comparison with that of the cases without MI (COV = 28.68 and SCV = 8.26). The findings for MI were confirmed by the sensitivity analysis considering cases with and without ACS, with the observed difference in variation between the two groups being less pronounced, and the measures of variation showed the highest values in cases with stable CHD or CP (see Additional file 1: Table A3).

**Table 2:** Crude rate, direct standardized rate (DSR) and measures of variation

	Cases	Cases without MI	Cases with MI
*Crude data*			
Number of CAs	425,163	349,621	75,542
Crude rate per 10,000 population	101.86	83.76	18.10
*Age-sex DSR in 401 districts per 10,000 population*			
Median	101.67	82.82	17.41
Mean (SD)	102.69 (25.98)	84.32 (24.18)	17.61 (3.44)
Min	46.79	27.08	9.58
Max	229.19	210.62	33.77
*Measures of variation*			
COV	25.29	28.68	19.53
SCV	6.44	8.26	3.37

CA, coronary angiography; COV, coefficient of variation; DSR, direct standardized rate; MI, myocardial infarction; SCV, systematic component of variation; SD, standard deviation

**Fig. 1:**
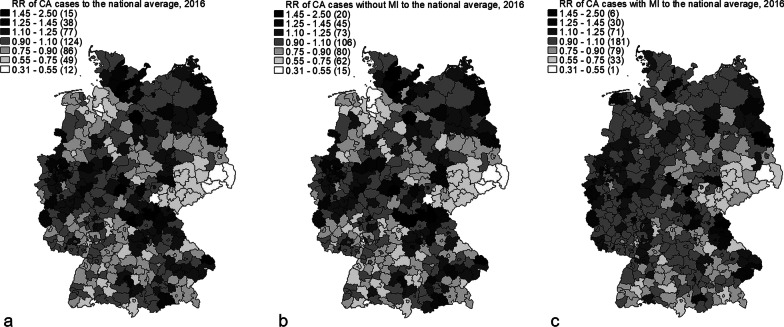
Map of CA **a** cases overall, **b** cases without MI and **c** cases with MI. Ratio of rates (RR) for the respective national average, 2016, classification method: custom; number in brackets displays the number of districts belonging to each class. *Source*: own depiction

**Fig. 2:**
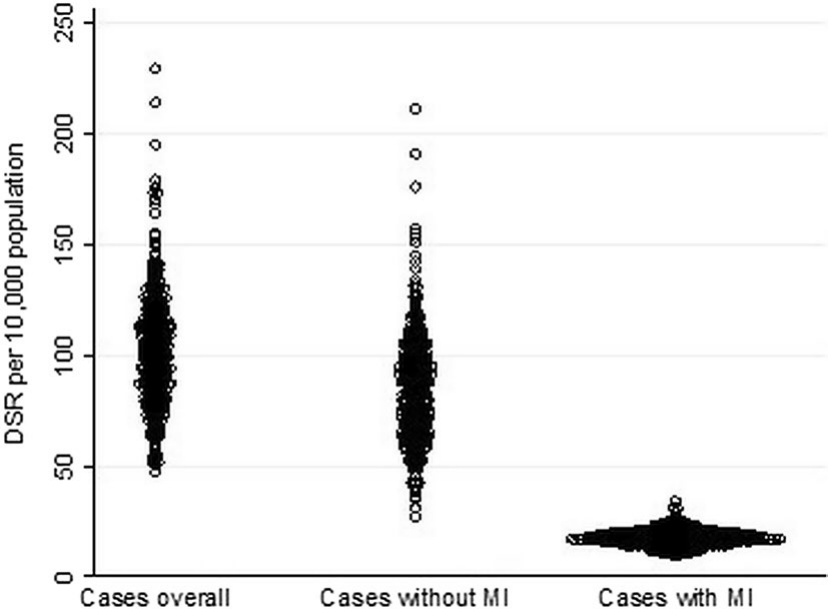
Direct standardized CA rate per 10,000 population by treatment diagnosis, 2016. Each dot represents one of the 401 districts

Figure 1 depicts the distribution of the CA rates of cases overall and cases with and without MI expressed as the ratio of rates (RR) to the respective national average across the districts of Germany. The distribution of high rates in overall cases showed a similar pattern compared with cases without MI, whereas cases with MI showed a different pattern. The maps also showed the wider distribution of the overall cases and the cases without MI in comparison with cases with MI. This is also illustrated in Fig. 2, which depicts the DSR for the three groups in the 401 districts per 10,000 population (see Additional file 1: Figure A1 for the DSR maps).

### Model results

The Lagrange Multiplier test verified the theoretical consideration regarding the model specification. Table 3 shows the regression results of Model A (SARE) and Model B (SCRARE) with the two specifications of the weight matrix, *Wr* and *We*. ρ, the estimated value of the spatial autocorrelation parameter, was positive and significant in all models, indicating moderate spatial autoregressive dependence in the error term. In other words, an exogenous shock to one district would cause moderate changes in the CA rate in the neighboring districts. The differences between both models and the matrix specifications were rather small. The specification of the weight matrix by means of eigenvalue-normalization (*We*) showed a higher Peusdo-R^2^ value compared with the row-normalization (*Wr*), signaling a slightly superior model fit. Estimation Model B, which included a spatial lag of the independent variable measuring the availability of hospitals with cardiac catheterization laboratory facilities, yielded similar direct effects in all patient subgroups. The coefficients of the cross-regressive effect of the availability of hospitals with a cardiac catheterization laboratory (W × Cath labs) were all nonsignificant, except for the CA rate of cases without ACS and cases with CHD and CP (see Additional file 1: Table A4). In terms of the explanatory power of the examined factors regarding the regional variations in the patient subgroups, both models and the weight matrix specifications arrived at similar conclusions. We therefore focused primarily on reporting the results of Model A because Model B, which included the spatial lag of the independent variable measuring the availability of hospitals with cardiac catheterization laboratory facilities, did not add substantial further information.

**Table 3:** Model results

	Cases overall				Cases without MI				Cases with MI			
	Model A (SARE)		Model B (SCRARE)		Model A (SARE)		Model B (SCRARE)		Model A (SARE)		Model B (SCRARE)	
	*Wr*	*We*	*Wr*	*We*	*Wr*	*We*	*Wr*	*We*	*Wr*	*We*	*Wr*	*We*
Observations	401	401	401	401	401	401	401	401	401	401	401	401
Pseudo R-squared	0.4186	0.4265	0.4261	0.4352	0.3813	0.3894	0.3895	0.3992	0.3986	0.4005	0.3995	0.4023
*β (SE)*												
Estimate of CHD prevalence^a^	10.01 (0.88)***	10.48 (1.63)***	9.87 (0.88)***	10.38 (0.88)***	8.99 (0.81)***	9.44 (0.81)***	8.87 (0.81)***	9.35 (0.81)***	1 (0.14)***	1.01 (0.14)***	0.99 (0.14)***	1 (0.14)***
GISD 2012	0.16 (0.1)	0.1 (0.11)	0.15 (0.1)	0.11 (0.1)	0.13 (0.09)	0.07 (0.09)	0.12 (0.09)	0.08 (0.09)	0.04 (0.02)**	0.04 (0.02)**	0.04 (0.02)**	0.04 (0.02)***
Cath labs^b^	40.62 (16.33)**	45.92 (19.83)**	53.41 (18.16)***	56.68 (18.32)***	37.64 (14.96)**	43.36 (15.77)***	49.34 (16.79)***	53.53 (16.95)***	2.91 (2.87)	2.97 (2.91)	3.35 (2.92)	3.49 (2.95)
W × Cath labs^b^			77.18 (47.71)	80.41 (49.84)			68.44 (44.18)	74.64 (46.56)			6.35 (7.3)	7.59 (7.14)
Constant	–143.81 (165.58)	–174.98 (142.44)	–167.02 (165.36)	–170.68 (173.94)	–103.5 (151.88)	–147.53 (160.93)	–123.82 (151.71)	–143.93 (160.46)	–20.05 (28.46)	–15.35 (28.81)	–21.27 (28.46)	–14.21 (28.78)
ρ	0.67 (0.05)***	0.72 (0.06)***	0.66 (0.05)***	0.71 (0.06)***	0.68 (0.05)***	0.72 (0.06)***	0.68 (0.05)***	0.72 (0.06)***	0.3 (0.06)***	0.31 (0.09)***	0.3 (0.06)***	0.31 (0.09)***

Coefficients for age and sex groups not reported (see Additional file 1: Table A1)

β, regression coefficient; GISD, German Index of Social Deprivation; MI, myocardial infarction; ρ, spatial autocorrelation parameter; SE, standard error

***p < 0.01, **p < 0.05, *p < 0.1

^a^Proportion of individuals in the total sample of insured with a confirmed diagnosis of CHD in 2016

^b^Per 10,000 population

With regard to Model A, the estimate of the prevalence of diagnosed CHD showed a strong positive association with the rates of CAs in the overall rate and all the patient subgroups and appeared to be a good measure for adjusting for the morbidity differences among the districts. An increase of 1% in the proportion of the population with a confirmed diagnosis of CHD in a district led to an estimated 10.01 additional CAs per 10,000 population (increase of approximately 10%) in the overall rate. The model showed a strong positive correlation between the number of hospitals with a cardiac catheterization laboratory per 10,000 population and the overall rate of CAs. One additional hospital with catheterization facilities per 10,000 was estimated to equate to 41–46 additional cases per 10,000 population, which corresponds to a 38–43% increase in overall cases. In contrast, no association was found in cases treated for MI, whereas cases without MI showed a strong positive correlation between the number of hospitals with a cardiac catheterization laboratory per 10,000 population and the rate of CAs. One additional hospital with catheterization facilities per 10,000 was estimated to equate to 38–43 additional cases without MI per 10,000 population, which amounts to a 43–50% increase in the rate. Our sensitivity analysis with ACS and stable CHD or CP cases showed similar results (correlation in cases without ACS and in cases with CHD or CP; no correlation in cases with ACS; see Additional file 1: Table A4). The GISD exhibited no significant association with the overall CA rate or cases without MI. In contrast, it showed a positive significant correlation with the CA rate in cases treated for MI (and ACS, see Additional file 1: Table A4).

In Model B, the coefficients of the cross-regressive effect of the availability of hospitals with a cardiac catheterization laboratory (W × Cath labs) show no significant association in the groups in the main analysis. However, the sensitivity analysis of cases without ACS in Model B showed a positive association between both the availability of hospitals with cardiac catheterization laboratory facilities in the districts and the spatially lagged variable for both weight matrix specifications. This finding suggests that, next to the local influence of supply, the available capacity in neighboring districts also affected the rates of CAs in this subgroup. In cases with stable CHD and CP, Model B with the eigenvalue-normalization weight matrix (*We*) indicated a positive association, whereas no significant association was found in the model with the row-normalized weight matrix (*Wr*) specification (see Additional file 1: Table A3).

## Discussion

Examining the rates of CAs in Germany based on a comprehensive set of nationwide routine data from statutory health insurances, we found substantial regional differences in utilization among the 401 districts. As we hypothesized, the observed variation differed among the subgroups based on the indication for treatment. In the case of MI (and ACS), the indication for CAs is unequivocal, and the benefits of intervention are well established. There is, consequently, comparably little variation in the observed rates of the procedure among the districts in these groups. In contrast, much higher variation was found in the cases without MI or ACS, and the highest variation was found in the rate of cases with stable CHD or CP.

In the spatial regression model that allowed for the spatial correlation of the error terms, we investigated the association of this variation in the rate among the districts and supply factors while controlling for need in a given locality. CHD morbidity showed a strong association with the regional rates of CAs in all the investigated subgroups. In addition, an association between regional deprivation and the rates of CAs was found in the acute cases, but no such association was seen in the other groups. By using a nationwide dataset and adding information extracted from the quality reports of the hospitals on the availability of hospitals with cardiac catheterization laboratory facilities per 10,000 population in the model, we were able to investigate the association between the regional health care supply and regional CA rates. Our findings suggest that after adjusting for the observable morbidity of the population, there was a positive correlation between the overall regional rates of CAs and the availability of hospitals with catheterization facilities. The analysis by subgroups based on the treatment diagnosis revealed a positive association between regional capacity and the rates of nonacute cases, whereas no such relationship was seen in the cases treated for MI (or ACS). The results supported the hypothesis that the association of need and supply factors differed by indication for CA, reflecting the differences with regard to the expected benefits and the uncertainty surrounding the treatment decision. In addition, the findings from the SCRARE model suggested that an indirect effect through the supply of neighboring districts might be at play in the subgroups of cases excluding ACS and possibly those with stable CHD or CP.

Piedmont et al. [18] studied the rate of utilization and the therapeutic consequences of CAs in the districts of the federal state of Saxony-Anhalt, Germany, and found that the considerable variation could not be explained by demographic differences. They also found that an almost linear association was seen between the regional frequency of CAs and the number of cases without therapeutic consequences within 12 months. The authors noted that they were unable to satisfactorily assess the influence of regional supply structures on the frequency of use because of a lack of data, but they pointed out that the wide range in the proportion of procedures without therapeutic consequences points toward an influence of supply-related factors. The relationship between the use of CA and regional capacity has been studied in the US [14]. The authors compared the association of per capita catheterization laboratories, per capita cardiologists and multi-provider markets with the utilization rates for CA in northern New England, USA. They found that variation in the use of the invasive cardiac procedure was strongly associated with the population-based availability of catheterization facilities and multi-provider markets and was unrelated to the supply of cardiologists or need. Our analysis revealed a similar relationship for the overall rate in Germany. In addition, we found that the relative association of the supply and need factors varied depending on the patient subgroup.

There may be reasons beyond population morbidity and supply that explain why the utilization of CA is higher in some districts than in others. For example, because the demand for the procedure is influenced by not only the actual need but also the perceived need, one explanation for the existing variations could be differences in patient preference. Patients sometimes appear to hold a more optimistic view of the marginal benefits of the treatment. For example, the COURAGE trials showed that for patients with stable angina, PCI provided no benefit in terms of survival or major cardiovascular events, although it did reduce pain and improve functioning [44]. In a matched survey of patients and physicians in one teaching hospital published by Rothberg et al. [45], physicians understood this evidence from the COURAGE trial; however, their patients falsely interpreted the results as a protective effect with regard to mortality and the risk of MI. Similarly, Kureshi et al. [46] concluded that patients had a poor understanding of the benefits of elective PCI, with significant variation across sites after conducting interviews with 991 patients with stable CHD undergoing elective PCI in 10 US academic and community hospitals. They therefore recommended that the anticipated benefits should be explained and discussed in detail before performing PCI. However, it is unlikely that the large variation observed across regions can largely be explained by individual patient preferences; instead, the variation is likely to be a combination of patient and physician preferences for a procedure. This is not ‘supplier-induced demand’ per se, but presumably, a patient forms beliefs of the expected marginal health benefit in part based on the expertise of their treating physician. Kureshi et al. [46] noted that wide variability existed in the ways in which hospitals obtained informed consent, and their findings suggested that hospital-level interventions in the structure and processes of obtaining informed consent for PCI might improve patient comprehension and understanding. Herwig and Weltermann [47] aimed to investigate patient-driven factors promoting suspected overuse in exploratory qualitative interviews with 25 patients suffering from CHD who had undergone at least one cardiac catheterization in two German teaching practices. The authors identified six patient factors that contributed to or prevented the use of catheterization: namely, the unquestioned acceptance of prescheduled appointments for procedures/convenience; disinterest in and/or lack of disease-specific knowledge; helplessness in situations with varying opinions on the required care; fear of cardiac events; the patient–physician relationship; and a patient’s experience that repeat interventions did not result in a change in health status or care. They concluded that most patients trusted their treating physicians’ recommendations for repeat CAs even if they were asymptomatic and that strategies to align physician adherence with guidelines and corresponding information to improve patient comprehension and understanding are needed.

## Limitations

Our findings suggest a strong association between CA rates and the supply of hospitals with catheterization facilities. However, this result does not provide causal evidence for supply-induced demand because we cannot establish the direction of causality based on regional data. Therefore, our regional analysis can only establish informed correlations. One reason for this could be that not only does supply influence the utilization of CA but also, conversely, more CHD patients in an area may attract more specialized physicians and facilities that can provide treatment in the region. If we presume supply to be endogenous, then any residual differences in regional health status that remain after adjustment for the observable factors will bias estimates of the impact of the availability of cardiac catheterization laboratories on CA rates. One objection to the presumption of endogeneity due to CHD patients attracting more specialized physicians is that there is no apparent association between the distribution of the regional supply and the estimate of the prevalence of diagnosed CHD. In addition, the overall rate and the rate of cases without MI (and without ACS and cases with stable CHD or CP) showed a positive association with the availability of hospitals with catheterization facilities; no such relationship was seen in the rate of cases with MI (and ACS). If the observed relationship between the rate of utilization and supply is indeed driven by the residual unobserved differences in CHD morbidity exerting an influence on regional capacity, there is no explanation as to why this association would not become apparent in the subgroups with MI (and with ACS). In contrast, the rate of cases with MI (and with ACS) showed a positive correlation with regional deprivation, possibly capturing the residual morbidity risks related to lifestyle. Nonetheless, caution is advised with regard to the interpretation of the relationship.

There are some other important limitations of the study. Although the available data on hospitals with catheterization facilities allow an assessment of the association with supply, the data did not contain information on office-based facilities, except for those listed in the reports as working in collaboration with a hospital. In addition, the number of catheterization laboratories within a given facility, details on the equipment and occupancy and the travel distance to the facilities were unknown. Such information could enhance the analysis and possibly contribute to a better understanding of the variation in the rates in the districts.

Additionally, the routine data from the statutory health insurances did not contain clinical data or information on symptoms and the results of noninvasive testing prior to the procedure. Such information would be a valuable addition for further investigation. Additionally, regional differences in the coding practice of diagnoses may exist, which could exert an influence on the classification of the patient groups. In some cases, cardiac computed tomography (CT) or cardiac magnetic resonance imaging (MRI) may be performed instead of invasive CA. However, unlike CA, cardiac CT and MRI are not yet part of the reimbursement catalog of services of the statutory health insurances in Germany and are not regularly reimbursed. Therefore, we assumed that these factors play a negligible role in the care of statutory health insurance patients and should have a very limited influence on the results of the study. Last, the local supply and use of noninvasive diagnostics before CA was not included as an observed variable in this analysis because some of these services could not be reliably mapped in the routine data. Therefore, we were unable to assess whether, and to what extent, structural deficits or problems with guideline adherence with respect to noninvasive diagnosis are associated with the observed regional variation.

## Conclusion

Our study highlighted large regional differences in the overall utilization of CA and different degrees of variation depending on the indication for the procedure. In addition, it demonstrated correlations between the overall rate and the regional health care supply while controlling for need in a given locality. Our findings for cases with and without MI suggested that, although regional differences in the rate of CAs in an acute situation are driven by need factors; in nonacute cases, supply factors also showed an association with utilization. Additionally, an association between regional deprivation and the rates of CAs was found in acute cases, but no such association was seen in the nonacute groups. Although our study can only establish informed correlations, it offers insight into patient access to and the provision of CA services, and can provide a platform for further local research to explain the mechanisms guiding regional variation in the use of CA in Germany.

## Supplementary Information

**Additional file 1**. Additional data and sensitivity analysis.

## Data Availability

The routine data analyzed in this study are available from the statutory health insurances TK, AOK and BARMER, but restrictions apply to the availability of the data, which were used under license for the current study and are not publicly available. To fulfill the legal requirements to obtain the data, researchers must obtain permission for a specific research question from the German Federal (Social) Insurance Office. Additionally, researchers must conclude a contract with the statutory health insurer regarding data access. The study must also be approved by the data protection officer both at the statutory health insurance and the research institute as well as the local ethics committee.

## Abbreviations

CA: Coronary angiography
SARE: Spatial‐autoregressive error models
SCRARE: Spatial cross-regressive models with autoregressive errors
MI: Myocardial infarction
PCI: Percutaneous coronary intervention
CABG: Coronary artery bypass grafting
SGB: Sozialgesetzbuch (Social Security Code)
ACS: Acute coronary syndrome
CHD: Coronary heart disease
EB: Empirical Bayes
OPS: Operationen- und Prozedurenschlüssel (Operation and Procedure Code)
EBM: Einheitlicher Bewertungsmaßstab (Uniform Value Scale)
CP: Chest pain
COV: Coefficient of variation
SCV: Systematic component of variation
ICD-10: International Statistical Classification of Diseases and Related Health Problems, 10th version
GISD: German Index of Social Deprivation
OLS: Ordinary least square
DSR: Direct standardized rate
RR: Ratio of rates
