# Supplementary material for: Regional variation in coronary angiography rates: the association with supply factors and the role of indication: a spatial analysis
Source: BMC Cardiovasc Disord. 2022 Feb 26;22:72. doi: 10.1186/s12872-022-02513-z (PMC8882285; doi:10.1186/s12872-022-02513-z)
Supplement: Supplementary file 1 — Additional file 1. Additional data and sensitivity analysis. [file 12872_2022_2513_MOESM1_ESM.pdf]

Appendix: Additional data and sensitivity analysis

Main analysis

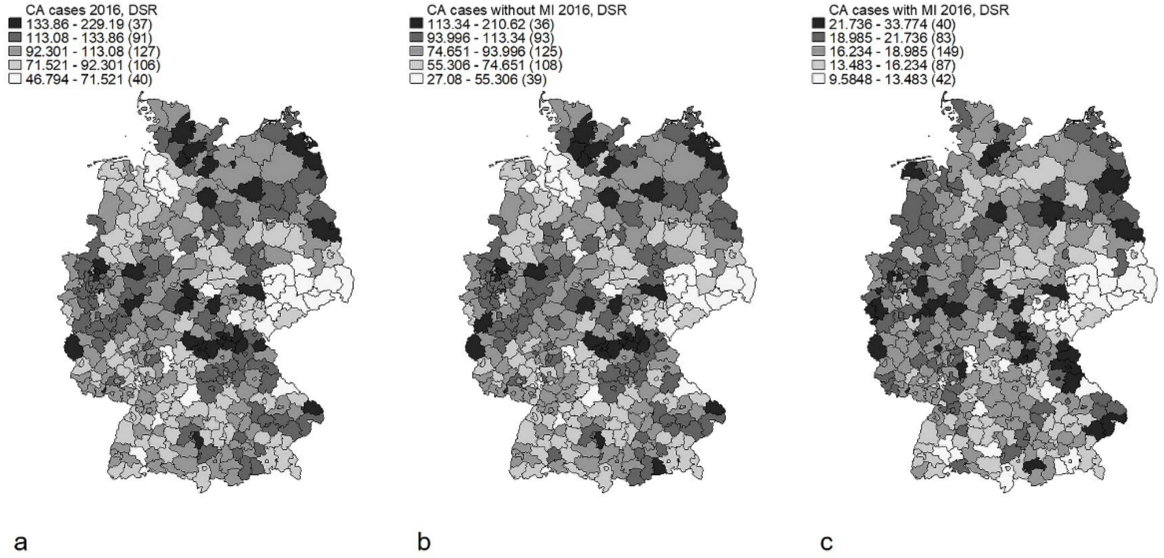

Figure A1 - Map of CA rate for (a) cases overall and (b) cases without MI (c) cases with MI, 2016; DSR, classification method: standard deviations; source: own depiction.

Table A1 – Model results of main analysis

	**Cases overall**				**Cases without MI**				**Cases with MI**			
	Model A (SARE)		Model B (SCRARE)		Model A (SARE)		Model B (SCRARE)		Model A (SARE)		Model B (SCRARE)	
	Wr	*We*	*Wr*	*We*	*Wr*	*We*	*Wr*	*We*	*Wr*	*We*	*Wr*	*We*
Observations	401	401	401	401	401	401	401	401	401	401	401	401
Pseudo R-squared	0.4186	0.4265	0.4261	0.4352	0.3813	0.3894	0.3895	0.3992	0.3986	0.4005	0.3995	0.4023
**β (SE)**												
Male under 40	185.89 (317.7)	226.79 (279.39)	221.69 (316.85)	220.09 (334.27)	110.03 (291.4)	176.18 (309.3)	141.32 (290.65)	170.21 (308.38)	41.98 (54.61)	32.46 (55.31)	43.86 (54.6)	31.17 (55.25)
Male 40 to 49	384.64 (690.36)	357.4 (685.16)	428.28 (687.63)	360.14 (715.09)	437.53 (634.6)	459.59 (662.78)	477.15 (632.15)	465.56 (660.73)	-118.57 (114.21)	-138.18 (114.61)	-118.46 (114.11)	-142.07 (114.49)
Male 50 to 59	-2024.69 (710.65)***	-2166.58 (742.87)***	-2109 (708.86)***	-2248.76 (731.19)***	-1933.22 (652.6)***	-2055.72 (675.33)***	-2009.34 (650.92)***	-2128.02 (674.8)***	-130.58 (119.5)	-139.88 (119.52)	-138.6 (119.76)	-153.38 (120.02)
Male 60 to 69	952.6 (841.23)	551.33 (1038.59)	1075.47 (839.54)	544.02 (875.41)	994.06 (773.41)	581.16 (811.5)	1108.82 (771.96)	578.01 (808.97)	3.08 (139.39)	4.93 (140.45)	9.17 (139.44)	1.47 (140.27)
Male 70 to 79	513.74 (864.64)	891.39 (1016.96)	385.25 (863.85)	755.05 (908.01)	149.08 (793.38)	565.48 (836.9)	37.41 (792.41)	443 (837.85)	243.73 (147.06)*	221.42 (148.35)	227.82 (148.1)	203.32 (149.08)
Male over 80	1727.12 (1226.06)	977.62 (1467.55)	1669.67 (1221.47)	1015.3 (1271.89)	1712.37 (1128.13)	1017.49 (1179.81)	1650.37 (1124.12)	1047.23 (1176.21)	66.65 (200.58)	27.56 (201.23)	74.85 (200.64)	40.26 (201.27)
Female 40 to 49	1345.16 (722.03)*	1329.83 (725.55)*	1389.8 (719.72)*	1311.1 (734.46)*	1121.11 (664.01)*	1100.2 (680.55)	1158.13 (661.88)*	1082.42 (678.53)	219.64 (119.03)*	224.08 (118.6)*	224.68 (119.08)*	222.01 (118.44)*
Female 50 to 59	1686.59 (851.19)**	2234.07 (931.69)**	1748.7 (847.81)**	2271.09 (892.37)**	1489.62 (780.36)*	2011.31 (825.2)**	1545.36 (777.26)**	2043.58 (822.95)**	224.61 (146.95)	240.62 (148.36)	229.38 (146.92)	246.76 (148.26)*
Female 60 to 69	-1006.93 (810.09)	-832 (947.13)	-1019.72 (806.48)	-836.8 (839.34)	-1103.98 (744.53)	-882.22 (777.6)	-1121.51 (741.22)	-891.23 (775.22)	28.18 (134.8)	9.91 (135.6)	31.43 (134.73)	14.41 (135.46)
Female 70 to 79	-368.72 (802.25)	-849.58 (941.33)	-248.38 (799.99)	-770.02 (855.93)	-102.24 (734.77)	-593.27 (789.97)	7.44 (732.69)	-519.64 (788.98)	-189.04 (141.39)	-181.01 (143.88)	-180.33 (141.6)	-173.18 (143.87)
Female over 80	-99.59 (697.38)	648.99 (825.48)	-38.35 (695.64)	691.95 (729.09)	-288.55 (641.09)	466.04 (675.73)	-235.08 (639.62)	507.65 (674.12)	23.2 (114.66)	29.87 (115.37)	24.01 (114.57)	28.88 (115.19)
Estimate of CHD prevalence[a]	10.01 (0.88)***	10.48 (1.63)***	9.87 (0.88)***	10.38 (0.88)***	8.99 (0.81)***	9.44 (0.81)***	8.87 (0.81)***	9.35 (0.81)***	1 (0.14)***	1.01 (0.14)***	0.99 (0.14)***	1 (0.14)***
GISD 2012	0.16 (0.1)	0.1 (0.11)	0.15 (0.1)	0.11 (0.1)	0.13 (0.09)	0.07 (0.09)	0.12 (0.09)	0.08 (0.09)	0.04 (0.02)**	0.04 (0.02)**	0.04 (0.02)**	0.04 (0.02)***
Cath labs[b]	40.62 (16.33)**	45.92 (19.83)**	53.41 (18.16)***	56.68 (18.32)***	37.64 (14.96)**	43.36 (15.77)***	49.34 (16.79)***	53.53 (16.95)***	2.91 (2.87)	2.97 (2.91)	3.35 (2.92)	3.49 (2.95)
W × Cath labs[b]			77.18 (47.71)	80.41 (49.84)			68.44 (44.18)	74.64 (46.56)			6.35 (7.3)	7.59 (7.14)
Constant	-143.81 (165.58)	-174.98 (142.44)	-167.02 (165.36)	-170.68 (173.94)	-103.5 (151.88)	-147.53 (160.93)	-123.82 (151.71)	-143.93 (160.46)	-20.05 (28.46)	-15.35 (28.81)	-21.27 (28.46)	-14.21 (28.78)
ρ	0.67 (0.05)***	0.72 (0.06)***	0.66 (0.05)***	0.71 (0.06)***	0.68 (0.05)***	0.72 (0.06)***	0.68 (0.05)***	0.72 (0.06)***	0.3 (0.06)***	0.31 (0.09)***	0.3 (0.06)***	0.31 (0.09)***

***p<0.01, **p<0.05, *p<0.1.
a: proportion of individuals in the total sample of insured with a confirmed diagnosis of CHD during the year 2016
b: per 10,000 population
β: regression coefficient, GISD: German Index of Social Deprivation, MI: myocardial infarction, ρ: spatial autocorrelation parameter, SE: standard error

Sensitivity Analysis

Table A2 - Frequency and characteristics of cardiac catheterization by treatment setting

	Total	Hospital		Office-based practice
		Inpatient hospital	Outpatient hospital	
**Cases**	n (column %)	N (row %)		
CA	425,163	369,882 (87.00%)	19,317 (4.54%)	35,964 (8.46%)
**Treatment diagnosis***				
Cases with ACS	111,892 (26,31%)	109,884 (98.21%)	67 (0.60%)	1,338 (1.20%)
Cases without ACS	313,271 (73,68%)	259,998 (82.99 %)	18,647 (5.95%)	34,626 (11.05%)
Cases with stable CHD or CP	167,696 (39,44%)	125,107 (74.60 %)	15,735 (9.38%)	26,854 (16.01%)

*inpatient main hospital diagnosis, outpatient hospital diagnosis, confirmed ambulatory diagnosis in treatment case
ACS: acute coronary syndrome, CA: coronary angiography, CHD: coronary heart disease, CP: chest pain

Table A3 - Crude rate, direct standardized rate (DSR) and measures of variation

	Cases without ACS	Cases with ACS	Cases with stable CHD or CP
**Crude data**			
Number of CA	313,271	111,892	167,696
Crude rate per 10,000 population	75.05	26.81	40.18
**Age-gender DSR in 401 districts per 10,000 population**			
Median	74.15	25.78	39.65
Mean (SD)	75.45 (22.41)	26.46 (6.44)	40.13 (13.28)
Min	24.18	12.03	9.70
Max	198.53	56.11	98.00
**Measures of variation**			
COV	29.70	24.33	33.08
SCV	8.85	5.64	10.64

ACS: acute coronary syndrome, CA: coronary angiography, CHD: coronary heart disease, COV: coefficient of variation, CP: chest pain, DSR: direct standardized rate, SCV: systematic component of variation, SD: standard deviation

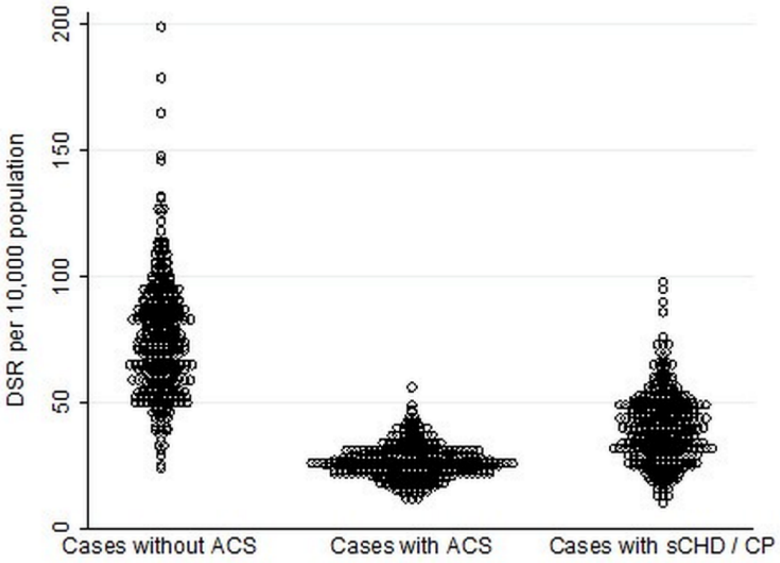

Figure A2 - Direct standardized CA rate per 10,000 population by treatment diagnosis, 2016.

Each dot represents one of the 401 districts.

Appendix Table A4 – Model results of sensitivity analysis

	**Cases without ACS**				**Cases with ACS**				**Cases with sCHD or CP**			
	Model A (SARE)		Model B (SCRARE)		Model A (SARE)		Model B (SCRARE)		Model A (SARE)		Model B (SCRARE)	
	Wr	*We*	*Wr*	*We*	*Wr*	*We*	*Wr*	*We*	*Wr*	*We*	*Wr*	*We*
Observations	401	401	401	401	401	401	401	401	401	401	401	401
Pseudo R-squared	0.3657	0.3727	0.3763	0.3845	0.3675	0.3702	0.3678	0.3708	0.2464	0.2464	0.2505	0.2612
**β (SE)**												
Male under 40	63.19 (272.65)	139.54 (290.3)	96.84 (271.77)	133.36 (289.17)	91.71 (93.84)	79.76 (95.6)	92.83 (93.91)	78.88 (95.59)	193.78 (168.08)	239.41 (178.52)	212.24 (167.81)	234.75 (177.45)
Male 40 to 49	443.38 (594.76)	495.68 (622.49)	483.44 (592.05)	504.99 (620.08)	-175.12 (197.14)	-210.81 (198.64)	-174.75 (197.13)	-213.96 (198.63)	155.21 (365.51)	228.71 (381.6)	173.38 (364.38)	231.37 (379.36)
Male 50 to 59	-1511.95 (611.14)**	-1691.86 (634)***	-1587.57 (609.09)***	-1766.01 (632.88)***	-453.16 (205.86)**	-439.55 (206.72)**	-457.72 (206.45)**	-449.27 (207.77)**	-860.57 (376.13)**	-960.83 (389.44)**	-900.57 (375.53)**	-1021.7 (388.1)***
Male 60 to 69	618.55 (724.96)	283.89 (762.22)	732.93 (723.11)	286.08 (759.25)	216.88 (240.43)	157.05 (243.32)	220.76 (240.74)	153.76 (243.28)	98.96 (445.42)	-112.78 (467.12)	155.86 (444.9)	-115.09 (464.38)
Male 70 to 79	176.09 (742.52)	559.58 (785.61)	62.38 (740.89)	434.24 (785.72)	266.21 (253.05)	280.09 (256.59)	257.45 (254.96)	266.66 (258.13)	192.23 (457.51)	471.04 (482.76)	127.09 (457.53)	373.08 (481.98)
Male over 80	1568.07 (1058.14)	859.85 (1108.54)	1500.74 (1053.72)	884.51 (1104.34)	283.65 (346.52)	229.45 (348.99)	287.53 (346.8)	238.99 (349.37)	1093.08 (649.36)*	811.32 (678.3)	1062.11 (647.42)	834.74 (674.49)
Female 40 to 49	849.2 (622.56)	829.17 (639.14)	888.32 (620.07)	809.2 (636.74)	498.01 (205.46)**	507.68 (205.35)**	500.75 (205.71)**	506.41 (205.3)**	755.97 (382.34)**	662.18 (391.92)*	781.57 (381.41)**	647.8 (389.66)*
Female 50 to 59	1167.86 (729.84)	1717.64 (774.38)**	1227.97 (726.41)*	1749.62 (771.52)**	522.09 (252.46)**	539.12 (256.34)**	524.83 (252.64)**	543.84 (256.45)**	895.89 (450.26)**	1239.65 (476.55)***	927.9 (448.97)**	1265.8 (473.8)***
Female 60 to 69	-636.89 (697.7)	-491.73 (730.2)	-651.84 (694.07)	-505.5 (727.37)	-291.95 (232.46)	-256.19 (234.85)	-290.56 (232.52)	-252.38 (234.86)	44.97 (428.88)	38.8 (448.01)	43.92 (427.33)	35.31 (445.35)
Female 70 to 79	-113.45 (686.7)	-541.33 (741.15)	-2.07 (684.27)	-463.41 (739.5)	-269.16 (242.27)	-310.4 (248.29)	-264.06 (242.86)	-304.84 (248.57)	-216.9 (424.23)	-455.83 (456.58)	-159.66 (423.55)	-397.68 (454.55)
Female over 80	-343.78 (600.84)	417.81 (634.82)	-284.99 (599.09)	462.87 (632.86)	73.33 (198.1)	93.3 (200.16)	74.06 (198.11)	92.78 (200.06)	-147.18 (369.24)	193.36 (388.67)	-113.96 (368.64)	225.32 (386.67)
Estimate of CHD prevalence[a]	7.82 (0.76)***	8.26 (0.76)***	7.7 (0.76)***	8.16 (0.76)***	2.13 (0.24)***	2.16 (0.24)***	2.12 (0.24)***	2.15 (0.24)***	3.94 (0.46)***	4.07 (0.47)***	3.88 (0.47)***	4 (0.46)***
GISD 2012	0.08 (0.09)	0.03 (0.09)	0.07 (0.09)	0.04 (0.09)	0.08 (0.03)***	0.08 (0.03)***	0.08 (0.03)***	0.08 (0.03)***	0.06 (0.05)	0.06 (0.05)	0.06 (0.05)	0.07 (0.05)
Cath labs[b]	37.72 (13.99)***	43.42 (14.79)***	50.44 (15.8)***	54.07 (15.93)***	2.67 (4.93)	3.04 (5.01)	2.95 (5.03)	3.47 (5.1)	15.43 (8.64)*	19.08 (9.12)**	21.93 (9.64)**	26.6 (9.71)***
W × Cath labs[b]			72.23 (41.62)*	77.58 (43.97)*			3.56 (12.69)	5.75 (12.45)			38.53 (25.33)	56.98 (26.32)**
Constant	-83.54 (142.11)	-131.65 (151.04)	-105.32 (141.86)	-128.09 (150.45)	-41.26 (48.9)	-36.15 (49.78)	-41.99 (48.96)	-35.35 (49.8)	-124.5 (87.6)	-148.23 (92.89)	-136.39 (87.59)	-145.17 (92.34)
ρ	0.68 (0.05)***	0.72 (0.05)***	0.68 (0.05)***	0.71 (0)***	0.34 (0.07)***	0.36 (0.1)***	0.34 (0.07)***	0.36 (0.1)***	0.63 (0.05)***	0.68 (0.06)***	0.63 (0.05)***	0.67 (0.06)***

***p<0.01, **p<0.05, *p<0.1.
a: proportion of individuals in the total sample of insured with a confirmed diagnosis of CHD during the year 2016
b: per 10,000 population
β: regression coefficient, GISD: German Index of Social Deprivation, MI: myocardial infarction, ρ: spatial autocorrelation parameter, SE: standard error
